# Iron Chelation Inhibits Osteoclastic Differentiation In Vitro and in Tg2576 Mouse Model of Alzheimer’s Disease

**DOI:** 10.1371/journal.pone.0139395

**Published:** 2015-11-17

**Authors:** Jun-Peng Guo, Jin-Xiu Pan, Lei Xiong, Wen-Fang Xia, Shun Cui, Wen-Cheng Xiong

**Affiliations:** 1 Department of Neuroscience & Regenerative Medicine, and Department of Neurology,Medical College of Georgia, Georgia Regents University, Augusta, Georgia, United States of America; 2 Charlie Norwood VA Medical Center, Augusta, Georgia, United States of America; 3 Department of Pathology, Changchun University of Chinese Medicine, Changchun, Jilin, China; 4 Department of Rheumatology, Union Hospital, Tongji Medical College, Huazhong University of Science and Technology, Wuhan, China; Faculté de médecine de Nantes, FRANCE

## Abstract

Patients of Alzheimer’s disease (AD) frequently have lower bone mineral density and higher rate of hip fracture. Tg2576, a well characterized AD animal model that ubiquitously express Swedish mutant amyloid precursor protein (APPswe), displays not only AD-relevant neuropathology, but also age-dependent bone deficits. However, the underlying mechanisms remain poorly understood. As APP is implicated as a regulator of iron export, and the metal chelation is considered as a potential therapeutic strategy for AD, we examined iron chelation’s effect on the osteoporotic deficit in Tg2576 mice. Remarkably, in vivo treatment with iron chelator, clinoquinol (CQ), increased both trabecular and cortical bone-mass, selectively in Tg2576, but not wild type (WT) mice. Further in vitro studies showed that low concentrations of CQ as well as deferoxamine (DFO), another iron chelator, selectively inhibited osteoclast (OC) differentiation, without an obvious effect on osteoblast (OB) differentiation. Intriguingly, both CQ and DFO’s inhibitory effect on OC was more potent in bone marrow macrophages (BMMs) from Tg2576 mice than that of wild type controls. The reduction of intracellular iron levels in BMMs by CQ was also more dramatic in APPswe-expressing BMMs. Taken together, these results demonstrate a potent inhibition on OC formation and activation in APPswe-expressing BMMs by iron chelation, and reveal a potential therapeutic value of CQ in treating AD-associated osteoporotic deficits.

## Introduction

Alzheimer’s disease (AD), the most common neurodegenerative disorder, affects 10% of all people over age of 65 years old. Osteoporosis, another age-associated common bone-degenerative disorder, is characterized by low bone mineral density (BMD) and microarchitectural deterioration of bone tissue [1]. Intriguingly, AD patients frequently have lower BMD and higher rate of hip fracture, compared with the same aged normal population [2, 3]. Several risk genes/loci identified in AD patients encode proteins critical for osteoclastic activation and/or bone-mass homeostasis. However, the pathological mechanisms of AD-associated osteoporosis remain largely unclear.

Amyloid precursor protein (APP) is a mendelian gene for early-onset AD. Mutations in APP (e.g., Swedish mutation) identified in AD patients favors APP cleavage to generate beta-amyloid (Aβετα40–42), a major culprit of AD [4, 5, 6]. To investigate mechanisms of AD-associated osteoporosis, we examined bone structure in Tg2576 mice, which express Swedish mutant APP (APPswe) under the control of hamster prion promoter. Tg2576 develops AD-relevant neuro-pathological deficits in an age-dependent manner. It also shows age-dependent osteoporotic deficits, including reduced trabecular bone-mass in young adult age and deteriorated bone tissue at older age [7]. The osteoclast (OC) differentiation and activation in Tg2576 mice are also age-dependent and biphasic, with a slight increase of OCs in young adult, but a marked decrease in older age of the mutant mice [7]. Intriguingly, expression of APPswe in osteoblast (OB)-lineage cells suppresses OB differentiation in vitro and in vivo [8]. These observations suggest that APPswe/Abeta may be one of the common denominators underlying pathogenesis of AD neuropathology as well as AD-associated skeletal deficits.

In addition to neurons, APP is widely expressed in many tissues, including OCs, OBs, and their precursor cells [bone marrow stromal cells (BMSCs) and BMMs][7]. Although APP’s physiological function remains largely unclear, several lines of evidence suggest that it may regulate iron metabolism. First, APP binds to metals (e.g., Zn2+ and Co2+), and metal binding to Abeta stabilizes Abeta deposition [9, 10]. Second, APP translation/expression is regulated by irons, as its mRNA contains an iron response element [9, 10]. Third, APP promotes iron exporter in neurons, likely due to its interaction with ferriportin (FPN) and its ferroxidase activity [10]. Note that FPN (a key iron exporter) is highly expressed in macrophages, including BMMs [11]. Iron metabolism is also critical for bone-mass homeostasis. Patients with iron over-load (e.g., hemochromatosis) often have osteoporosis [12, 13] and patients with iron-deficiency anaemia have reduced bone remodeling and deleterious bone formation [14]. In culture, ferric ion facilitates OC genesis and bone resorption, and inhibits OB formation, likely by increased production of reactive oxygen species (ROS)[15]. However, whether APP/APPswe regulates iron metabolism in bone cells and how iron metabolism affects bone homeostasis remain poorly understood.

Iron chelation is considered as a possible agent in AD treatment. Clinoquinol (CQ, 5-chloro-7-iodo-quinolin-8-ol), a derivative of 8-hydroxyquinoline, is a moderate chelator for zinc (Zn2+), copper (Co2+), and iron (Fe2+). CQ has been found to be effective in treating AD-relevant neuropathology in various AD animal models, including Tg2576. Oral treatment of CQ in Tg2576 mice for 9 weeks reduces brain Abeta deposition by 40%, and rescues memory impairment [16]. However, the CQ’s function in bone is poorly understood.

Here, we use Tg2576 mouse model to examine CQ’s effect on AD-associated bone deficit. Oral treatment of CQ in young adult Tg2576 mice attenuated the bone-loss deficit. The CQ’s effect appears to be more potent in Tg2576, compared with that of WT (C57/BL6) mice. Interestingly, in vitro studies also show more potent CQ’s inhibitory effect on OC-genesis from APPswe-expressing BMMs. Low doses of CQ as well as DFO, another iron chelator, inhibit OC maturation, without an obvious effect on OB-differentiation. These results thus demonstrate a selectively inhibitory effect of iron chelation on OC genesis and activation, and an enhanced CQ/DFO’s iron chelation effect by expression of APPswe in BMMs.

## Materials and Methods

### Reagents and animals

The rabbit polyclonal antibodies used in this project include APP (Cell Signaling, Danvers, MA, USA), ferriportin (Thermo, Inc., Rockford, IL, USA), NFATC1 (BD biosciences, San Jose, CA, USA), phospho-Erk1/2 (pErk1/2) and phospho-Akt (pAkt)(Cell Signaling, Danvers, MA, USA). Murine macrophage colony-stimulating factor (M-CSF) was obtained from R&D Systems (Minneapolis, MN, USA), and the recombinant GST-RANKL proteins were provided by Dr. Xu Feng (University of Alabama at Birmingham), which were generated and purified as previously described [7, 17, 18].

As described previously[19], the Tg2576 mice, purchased from Taconic, Inc. (Hudson, NY, USA), express human APP695 with double mutations at KM670/671NL (Swedish mutations)(called APPswe) under the control of hamster prion promoter. Tg2576 has been crossed into C57BL/6 genetic background for more than 6 generations. All animal experimental procedures were approved by the Institutional Animal Care and Use Committee (IACUC) at the Georgia Regents University, in accordance US National Institutes of Health guidelines.

### In vivo CQ treatment

Both WT and Tg2576 mice were used, and each genotype was divided into two subgroups: A subgroup received drinking water, and B subgroup received drinking water containing CQ for a period of 3 months. Thus, total of 4-groups of mice, and 5 male mice per genotype group were tested. CQ in water (0.012%) was prepared and given to mice. The drinking water with or without CQ was changed every other day. ~4–5 ml of water was consumed by each mouse per day, which gives a cumulative dose ~ 30 mg/kg per mouse per day. We chose this dose based on previous publication (20). The CQ treatment was started at age of 1-month old in Tg2576 and WT mice. Mice were euthanized 3 months after the treatment. Long bone samples (e.g., femur) were collected for histomorphometric and Micro-computed tomography (μιχροCT) analyses, and mouse sera were collected for ELISA analysis of osteocalcin and RIA analysis of PYD levels.

### Micro-computed tomography (μιχροCT)

The microCT analysis was carried out as described previously [17, 18]. In brief, the femur samples (both the distal trabecular bone and midshaft cortical bone) were measured by Scanco microCT 40 (Scanco Medical AG, Brüttisellen, Switzerland) as detailed described previously [17, 18].

### Bone histomorphometric analysis

Bone histomorphometric analyses were carried out as previously described [17, 18]. In brief, the tibia and femurs were fixed overnight in 10% buffered formalin, and decalcified in 14% EDTA. They were then embedded in paraffin, and sectioned. The bone sections were subjected for H & E and Goldner’s Trichrome stain analyses, and counter stained by fast green. Bone histomorphometric perimeters were determined as previously described [17, 18], by measuring the areas situated at least 0.5 mm from the growth plate and excluding the primary spongiosa and trabeculae connected to the cortical bone.

### Measurements of serum levels of osteocalcin and deoxy-pyridinoline (PYD)

The ELISA analysis of osteocalcin and the RIA analysis of PYD were carried out as described previously [17, 18]. In brief, the mouse serum levels of osteocalcin were measured by use of mouse osteocalcin Elisa kit (Biomedical Technologies, Inc.), and the serum levels of PYD were determined by use of METRA Serum PYD EIA kit (QUIDEL Corporation). Both assays were carried out per instructions. All the ODs measured after reactions were converted to osteocalcin/PYD concentration according to their standard curves. All the samples were measured in duplicates.

### In vitro BMM and OC cultures

Mouse BMMs and OCs were generated as described previously [7, 17, 18]. In brief, the bone marrow cells were flushed out from femurs and tibiae of WT and Tg2576 mice (2–3 month old) with ice-cold alpha-MEM. The cells were plated on 100 mm tissue culture plates in alpha-MEM containing 10% FBS and 10 ng/ml recombinant M-CSF, and incubated at 37°C with 5% CO2 overnight. Non-adherent cells were harvested and subjected to Ficoll-Hypaque gradient centrifugation for BMM isolation. To induce osteoclastogenesis, 5×104 BMMs were incubated with OC differentiation medium containing recombinant M-CSF (10 ng/ml) and GST-RANKL (100 ng/ml) for various days. TRAP (tartrate-resistant acid phosphatase) staining analysis was used to confirm the OC identity.

### In vitro BMSC and OB cultures

Mouse BMSCs and OB were generated as described previously [19]. As described above, the bone marrow cells that were flushed out from long bones of 2–3 months old WT and Tg2576 mice were plated onto 100 mm tissue culture plates in DMEM containing 10% FBS (fetal bovine serum) and 1% penicillin/streptomycin (P/S). The adherent cells (BMSCs) were harvested and plated in a density of 1*104/cm2 for OB differentiation. To induce OB-differentiation, the BMSCs were maintained in osteogenic medium (DMEM containing 10% FBS, 1% P/S, 10 mM beta-glycerophosphate and 50μM L-Ascorbic Acid-2- phosphate) for 7–14 days. ALP (alkyline phosphatase) staining and quantification analyses were performed to confirm the OB differentiation as described previously [19].

### In vitro CQ and DFO treatments

CQ and DFO treatments were carried out as described previously [20, 21, 22]. In brief, 10 microM CQ and DFO were dissolved in DMSO as stock solutions. BMMs from WT and Tg2576 mice were cultured onto 35 mm tissue culture plates or 24-well plates and treated with different doses of CQ or DFO in the presence of RANKL with 1% of M-CSF. The OC identity was verified by TRAP staining analysis. The signaling events induced by RANKL or M-CSF were determined by Western blot analyses of lysates of treated cells.

### Western blot analysis

Western blot analysis was carried out as previously described [7, 17, 18]. The cell lysates were separated by 10% SDS polyacrylamide gel electrophoresis, and transferred to a PVDF membrane by electrotransfer for 1.5 h. The membrane was blocked with 5% skim milk in TBS-T, and then incubated with first antibodies overnight at 4°C. This procedure was followed by incubation with a horseradish peroxidase-conjugated secondary antibody for 1 hour. Chemiluminescence was detected by using an ECL system (GE Healthcare). The intensity of the band was scanned and analyzed.

### Measurement of intracellular iron levels

The total intracellular iron levels were measured by using BioVision's Iron Assay Kit (BioVision Inc). In this assay, ferric carrier protein will dissociate ferric into solution in the presence of acid buffer. After reduction to the ferrous form (Fe2+), iron reacts with Ferene S to produce a stable colored complex and give absorbance at 593 nm. Different concentrations of iron standard in a 96-well plat were also measured. According to the standard curve, the OD values of test samples were converted to their iron concentrations.

### Immunostaining analysis

BMMs plated on coverslips in 24-well plates were washed with PBS, and then fixed with 4% paraformaldehyde for 15 min. Cells were permeabilized with 1% Triton X-100 in PBS for 10 minutes at room temperature and rinse well with PBS. After blocking non-specific antibody binding sites by incubating cells for 10 min in blocking buffer (PBS + 0.1% Tween+ 1% serum), cells on the coverslips were incubated with indicated primary antibodies for overnight at 4°C. The coverslips were washed three times in PBS for 5 min each, and then incubated with fluorophore-conjugated secondary antibodies for 60 min at room temperature. The coverslips were mounted and subjected to imaging analysis by a confocal microscope, LSM510 (Zeiss), use of the 20X/05 EC Pla-Neofluar objective or the 63X/1.4 Oil Plan-Apochromat objective. The quantification was performed using Image-Pro Plus software (MediaCybernetics).

### Statistical analysis

All data were expressed as mean ± SD. The significance level was set at P < 0.05. Student t-test was used for statistical analysis.

## Results

### CQ-increase of bone-mass in Tg2576 mice

**Fig 1:**
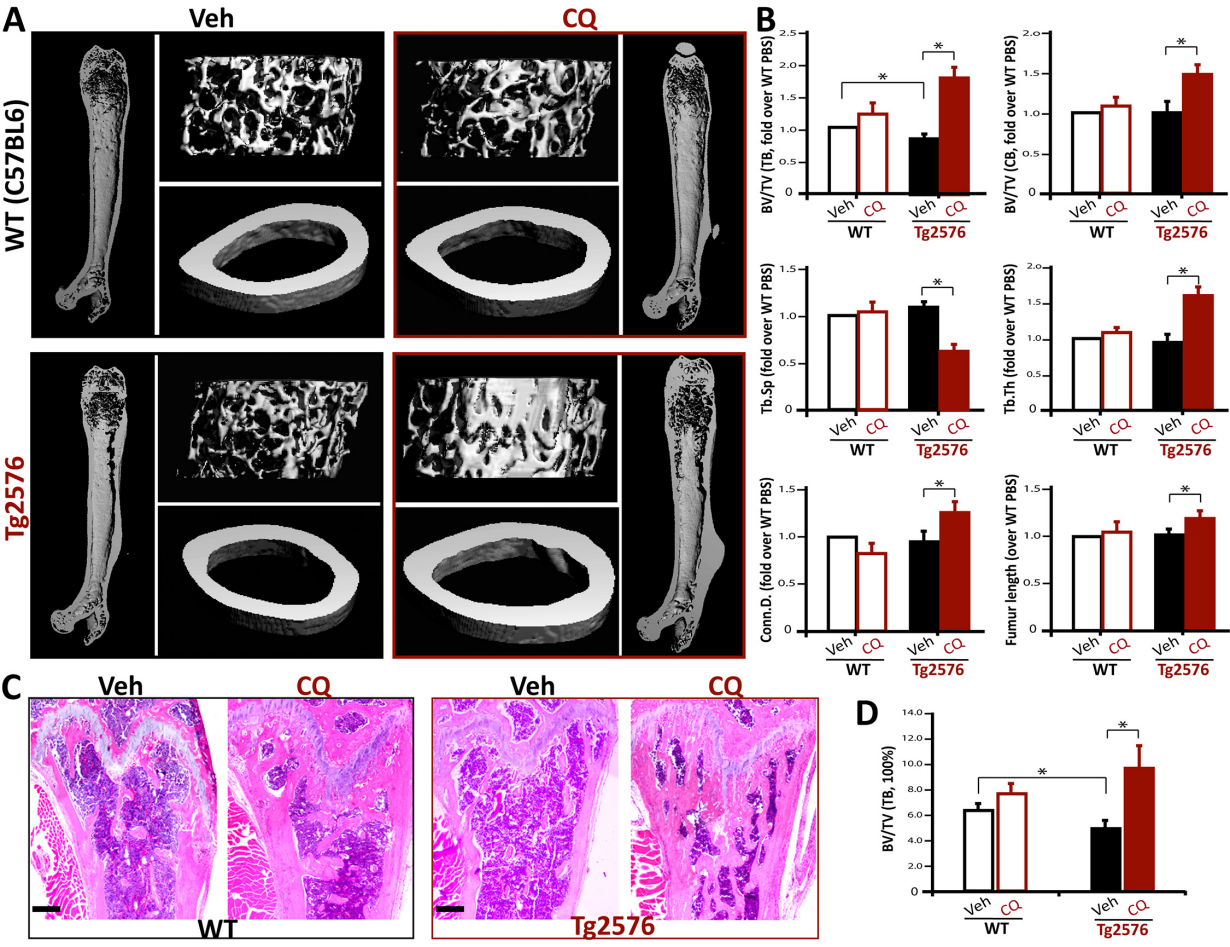
CQ restore of osteoporotic deficit in Tg2576 mice. Tg2576 mice (at 1 month old, 5 per group, male) were fed with drinking water containing with vehicle (Veh.) or CQ (30 mg/kg/day) for 3 months, and their femur bone samples were collected for phenotypic analysis. (A, B) The microCT analysis displayed an increased trabecular and cortical bone volumes of CQ-treated Tg2576 femurs. In addition, the trabecular bone thickness (Tb.Th) and trabecular separation (Tb.Sp) were all ameliorated by CQ treatment. The trabecular connectivity density (Conn.D.) and femur length were also improved by CQ. (C,D) Histomorphological examinations by hematoxylin and eosin (H & E) staining analysis showed an increased osteoid numbers of trabecular bones in CQ-treated Tg2576 femurs. Representative images were shown in C. Bars, 500 microm. In B and D, the values of mean ± SD from 5 different animals per genotype are shown. *, p<0.05, significant difference.

Iron chelation by CQ is found to be effective in restoring AD-like neurological deficit in AD animal models [20, 23]. We thus asked if CQ is useful in treating the osteoporotic deficit in Tg2576 mouse model of AD. Tg2576 and WT (C57BL6) mice (at age of 1-month old) were fed with drinking water containing vehicle (PBS) or CQ (30 mg/kg/day) for 3-months, and their femur bone samples and sera were then collected for phenotypic analysis. Remarkably, increased trabecular and cortical bone volumes were detected in CQ-treated Tg2576 femurs, as compared with that of vehicle controls, by micro-CT analysis (Fig 1A and 1B). The trabecular thickness (Tb. Th), trabecular connectivity (Conn. D.), and the femur length were all increased in CQ-treated Tg2576 mice (Fig 1A and 1B). To our surprise, CQ-increased bone-mass was more obvious in Tg2576, compared with that of WT mice (Fig 1A and 1B). In agreement with microCT analysis, histomorphological examinations of femur samples by H & E staining also displayed more increase of trabecular bone volumes in CQ-treated Tg2576 than that of WT mice (Fig 1C and 1D). These results thus demonstrate an efficient amelioration of the osteoporotic deficit in Tg2576 mice by CQ treatment.

### CQ-reduction of TRAP^+^ OCs and bone resorption in Tg2576 mice

**Fig 2:**
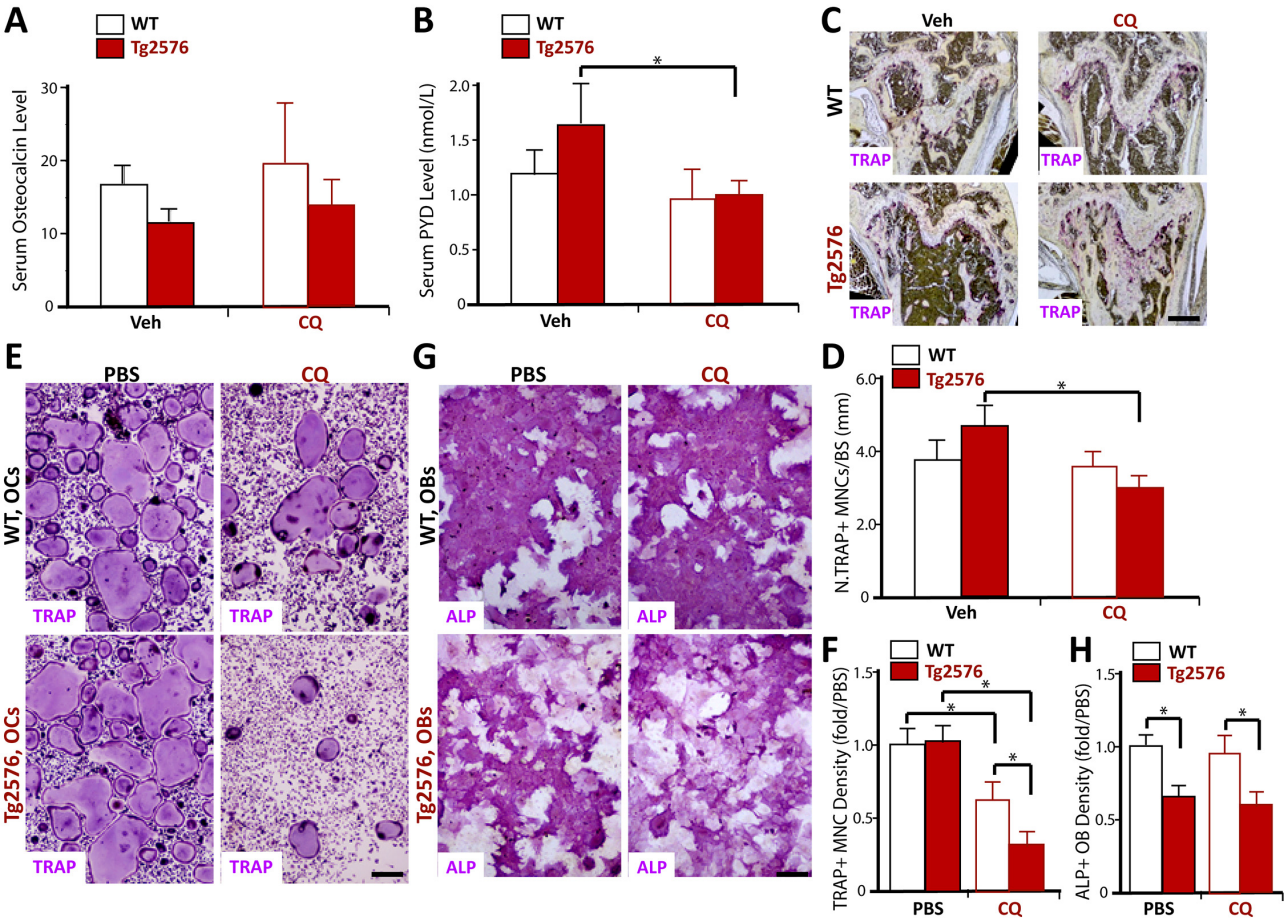
CQ inhibition of bone resorption and OC genesis. (A-B) Measurements of serum levels of osteocalcin (A) and PYD (B) in 4-month-old WT and Tg2576 mice treated with vehicle (Veh.) or CQ (for 3 months) by ELISA and RIA analyses, respectively. Five males per genotype were measured. (C-D) TRAP staining analysis of femur sections. C, Representative images. D, The quantitative analysis of TRAP+ cells per unit bone surface (BS) was carried out in trabecular bones of femurs. The values of mean ± SD from 5 different animals were shown. (E-H) Effects of CQ on in vitro OC and OB genesis from BMMs or BMSCs, respectively. E, Representative images of TRAP staining. F, Quantitative analyses of the average TRAP-positive multi-nuclei cell (MNC) density (count TRAP+ MNCs [>3 nuclei per cell] per unit area). G, Representative images of ALP staining at day 7 cultures. H, Quantitative analysis of average ALP activity (ALP positive area/over total area). The values of mean ± SD from 3 separate cultures were shown. *, p<0.05, significant difference.

The CQ increase of bone-mass may be due to CQ enhancement of bone formation and/or CQ reduction of bone resorption. To address this question, we measured serum levels of osteocalcin and PYD in WT and Tg2576 mice treated with or without CQ. The serum osteocalcin, a marker for bone formation, was lower in Tg2576 mice, compared with WT controls (Fig 2A), in line with our previous publication [8]. Upon CQ treatment, no obvious change in serum ostecalcin levels was detected between WT and Tg2576 mice (Fig 2A), suggesting a minimal role of CQ in bone formation. In contrast, the serum levels of PYD, a collagen cross-link molecule that provides valuable information on bone resorption, were decreased in Tg2576, but not WT mice, after CQ treatments, compared with that of vehicle treatments (Fig 2B). These results thus suggest a selective inhibition of bone resorption in Tg2576 mice by CQ, providing a potential underlying mechanism for CQ-increase of bone-mass.

The CQ decrease of bone resorption could result from a reduction in OC number and/or function. We thus examined TRAP+ OCs in CQ-treated Tg2576 and WT mice. The number of TRAP+ OCs per unit of bone surface was slightly lower in CQ-treated Tg2576 femurs, compared with the vehicle controls (Fig 2C and 2D). The reduction of TRAP+ OCs was undetectable in WT mice treated with CQ (Fig 2C). We then examined CQ’s effect on in vitro OC and OB differentiation (see Methods). At concentration of 5 microM, CQ reduced TRAP+ multi-nuclei cell (MNC) density in BMM cultures from Tg2576 mice (Fig 2E and 2F), without an obvious effect on ALP (alkaline phosphatase) activity, a marker for OB differentiation, in Tg2576-BMSC cultures (Fig 2G and 2H). Note that CQ also reduced TRAP+ cell density in WT-BMM cultures, but, its inhibitory effect was less potent than that in Tg2576-BMM cultures (Fig 2E and 2F). These results suggest that CQ acts as an inhibitor of OC genesis in culture and in vivo, and its inhibitory effect appeared to be more potent in Tg2576 or APPswe-expressing BMMs.

### Dose-dependent inhibition of in vitro OC-genesis by CQ

**Fig 3:**
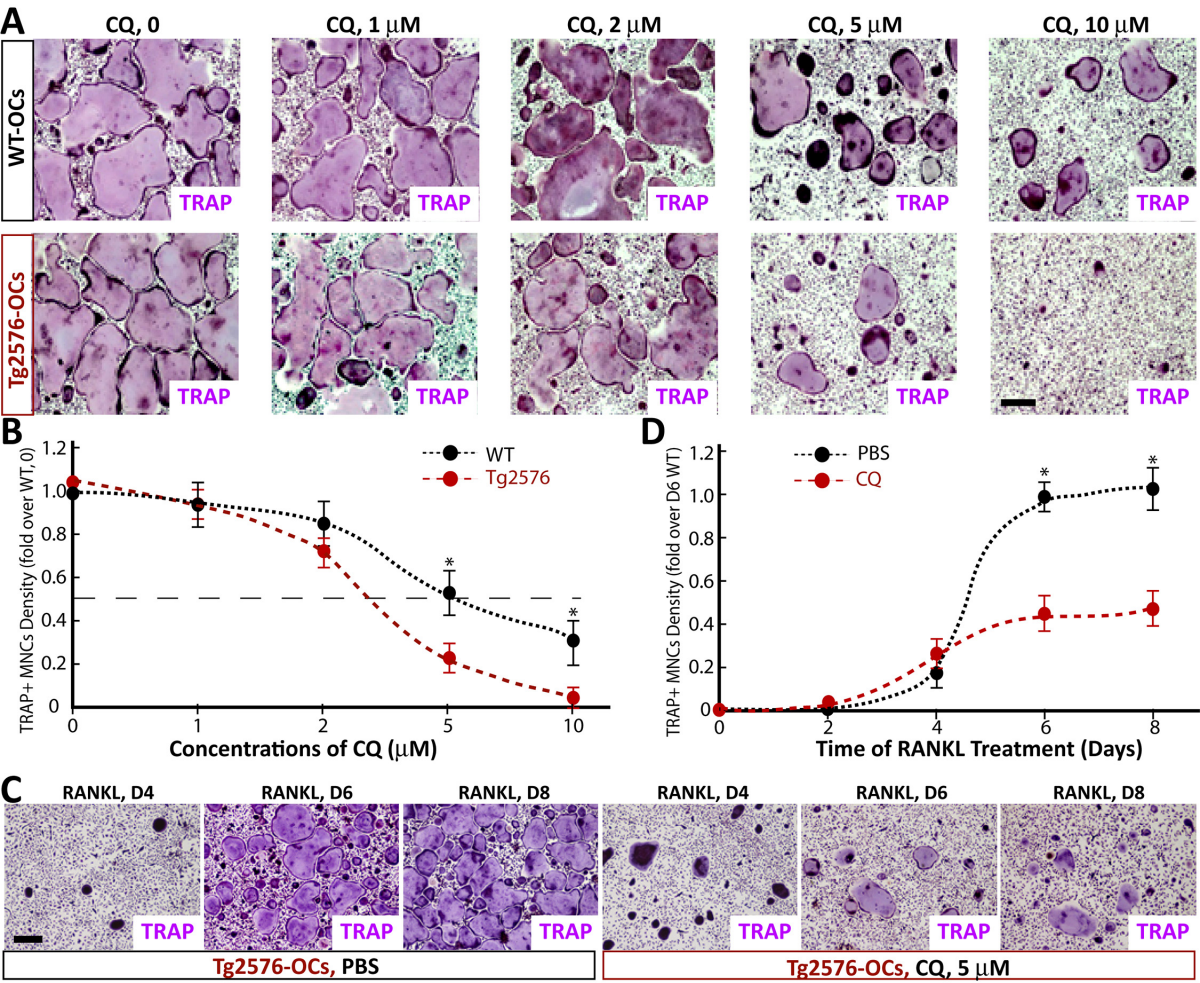
CQ suppression of in vitro OC genesis in a dose- and time-dependent manners. (A-B) BMMs from WT and Tg2576 mice were induced for OC differentiation in the presence or absence of various concentrations (1, 2, 5, and 10 microM) of CQ. At day 5 of RANKL induction, cells were stained for TRAP. A, Representative images. B, quantitative analyses. The effective concentrations of CQ for 50% inhibition of OC genesis is ~5 microM and ~2.25 microM in WT- and Tg2576-BMMs, respectively. (C-D) BMMs from WT and Tg2576 mice were induced for OC differentiation in the presence of CQ (5 microM). Cells were cultured for different days (day 6-D6, D8) and stained with TRAP. C, Representative images. D, Quantitative analyses. The values of mean ± SD from 3 separate cultures were shown. *, p<0.05, significant difference.

We then asked whether CQ-inhibition of OC genesis is dose-dependent. BMMs derived from untreated WT and Tg2576 mice were incubated with M-CSF and RANKL in the presence of various concentrations of CQ (1 to 10 microM). As shown in Fig 3A and 3B, CQ reduced TRAP+ MNC density in both WT and Tg2576 cultures in a dose-dependent manner. Again, a more potent CQ-inhibitory effect was detected in Tg2576-BMM cultures than that of WT-BMMs (Fig 3A and 3B). At ~2.25-microM concentration of CQ, a 50% reduction of TRAP+ MNCs was detected in Tg2576-BMM cultures, but little effect was observed in WT-BMM cultures (Fig 3A and 3B).

**Fig 4:**
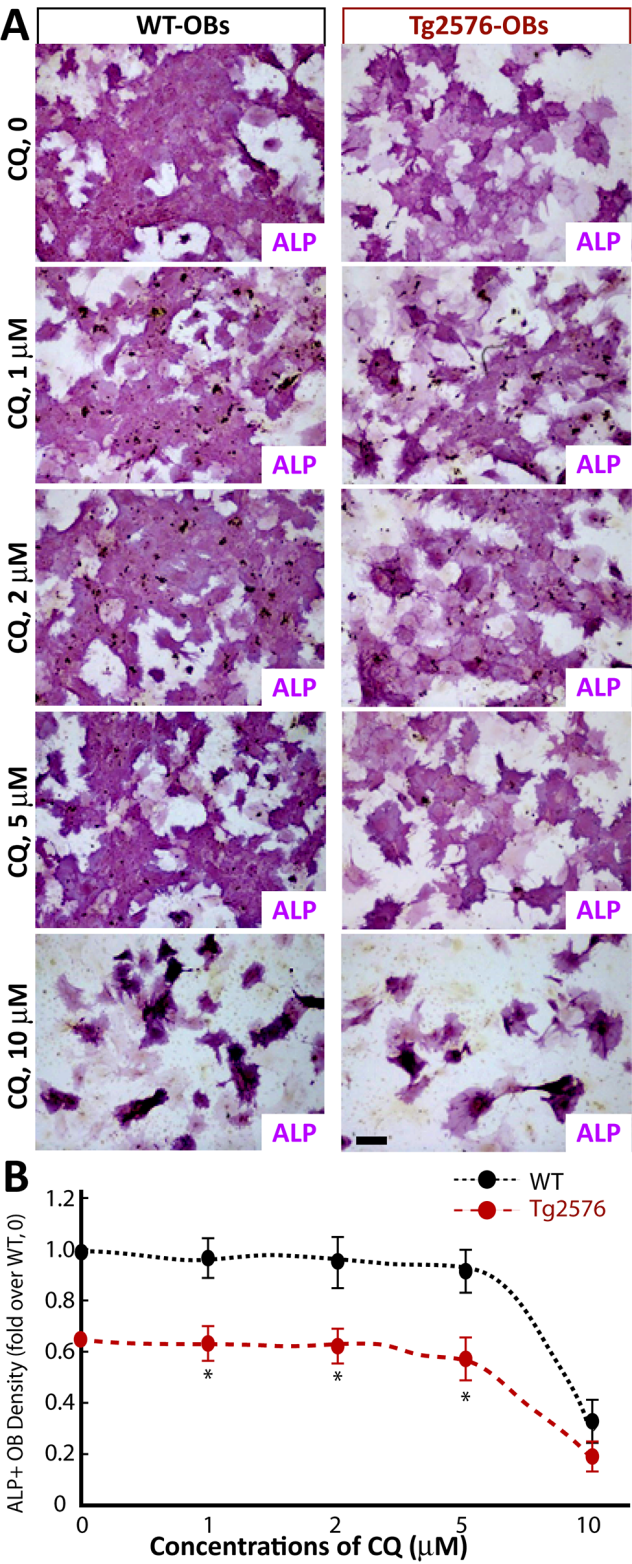
CQ effects on in vitro OB genesis. BMSCs from WT and Tg2576 mice were induced for OB differentiation in the presence of various concentrations of CQ. At day 7, cells were stained for ALP as described in the Methods. A, Representative images. B, Quantitative analyses. Note that lower concentrations (<5 microM) of CQ had little to no effect on ALP activity or OB differentiation. The values of mean ± SD from 3 separate cultures were shown.

We also tested CQ’s dose-dependent effect on OB differentiation by in vitro OB genesis assay. BMSCs from untreated WT and Tg2576 mice were incubated with OB differentiation medium in the presence or absence of various concentrations of CQ (see Methods). Consistent with previous publications [8], Tg2576-BMSC culture showed a reduction in ALP activity (Fig 4). It is of interest to note that CQ at low concentrations that inhibited OC genesis (e.g., 5 microM) had little to no effect on ALP activity or OB differentiation (Fig 4). Only at high concentration (>10 microM), CQ could reduce ALP in both WT and Tg2576-BMSC cultures (Fig 4). In aggregates, these results suggest that whereas high concentration of CQ inhibits both in vitro OC and OB differentiation, the low concentration of CQ suppresses OC-genesis that is enhanced by APPswe expression in BMMs.

### Inhibition of in vitro OC-genesis by DFO, another iron chelator

**Fig 5:**
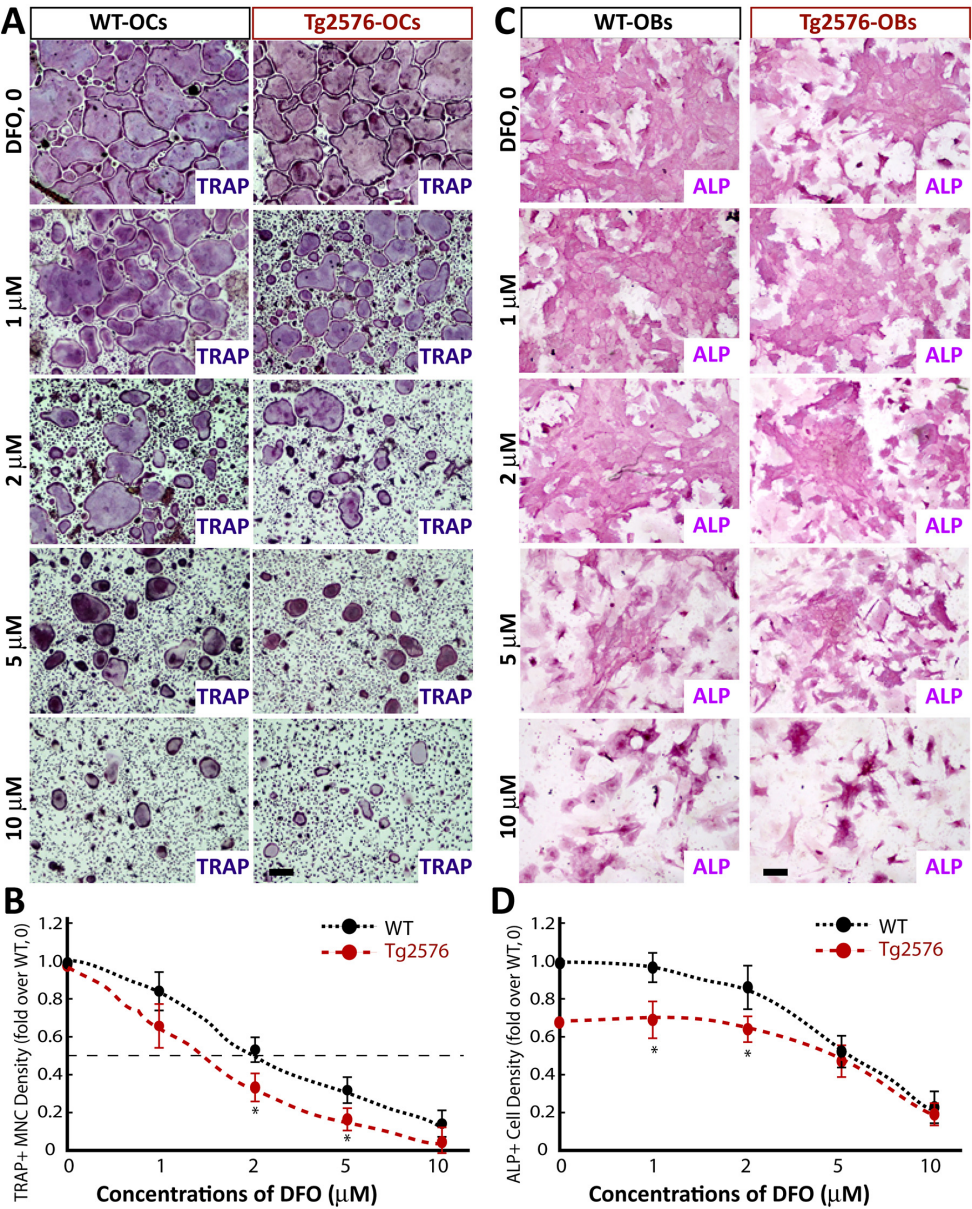
DFO inhibition of in vitro OC genesis in a dose-dependent manner. (A-B) BMMs from WT and Tg2576 mice were induced for OC differentiation in the presence or absence of various concentrations (0, 1-, 2-, and 5-μM) of DFO. At day 5 of RANKL induction, cells were stained for TRAP. A, Representative images. B, quantitative analyses. The effective concentrations of DFO for 50% inhibition of OC genesis is ~2-μM and ~1.5 microM in WT- and Tg2576-BMMs, respectively. (C-D) BMSCs from WT and Tg2576 mice were induced for OB differentiation in the presence of various concentrations of DFO. At day 7, cells were stained for ALP as described in the Methods. C, Representative images. D, Quantitative analyses. The values of mean ± SD from 3 separate cultures were shown.

As CQ is known to be a moderate iron chelator [21], we wondered whether other iron chelators have a similar effect as CQ on OC genesis. DFO, a powerful iron chelator, was thus tested. As shown in Fig 5A and 5B, DFO reduced TRAP+ MNC density in BMMs isolated from both WT and Tg2576 mice in a dose-dependent manner, as CQ did. DFO also showed a more potent inhibitory effect in BMM cultures from Tg2576 mice than that from WT mice (Fig 5A and 5B). Additionally, DFO at low concentrations (< 2 microM) had little effect on ALP activity during OB differentiation (Fig 5C and 5D). These results reveal a similar, but more potent, effect of DFO as that of CQ in suppression of both OC and OB differentiation in cultures, arguing for a potent inhibition of OC genesis by iron chelation.

### Normal M-CSF-induced signaling, but impairment of RANKL-driven Akt phosphorylation and NFATC1 activation, in CQ-treated BMMs

**Fig 6:**
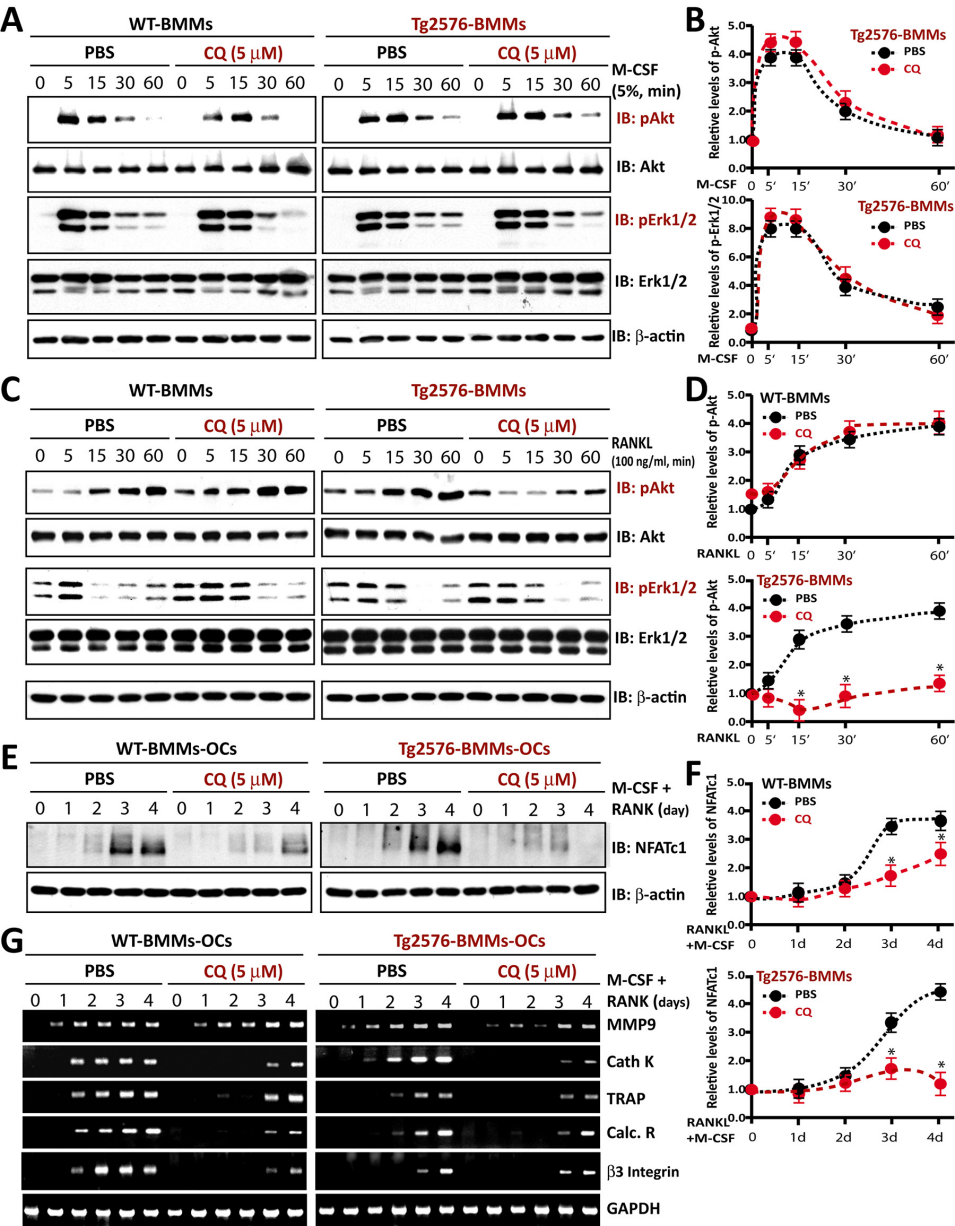
CQ effects on M-CSF- and RANKL-induced signaling in WT- and Tg2576-BMMs/pre-OCs in vitro. (A-B) CQ treatment had little to no effect on M-CSF-induced pErk1/2 and pAkt signaling in WT- and Tg2576-BMMs. A, Representative blots. B, Quantification analysis. The values of mean ± SD from 3 separate cultures were shown. (C-D) RANKL-induced Akt phosphorylation in Tg2576-BMMs, but not in WT-BMMs, was impaired by CQ treatment. C, Representative blots. D, Quantification analysis. The values of mean ± SD from 3 separate cultures were shown. *, P < 0.05, significant difference. (E-F) CQ reduced activation of the NFATc1 in both WT- and Tg2576-BMMs/pre-OCs. E, Representative blots. F, Quantification analysis. Note that a more dramatical reduction of NFATc1 activation by CQ was detected in Tg2576-BMMs-OC cultures. The values of mean ± SD from 3 separate cultures were shown. *, P < 0.05, significant difference. (G) RT-PCR analysis also showed reduced expression of genes associated with OC maturation, such as MMP9, calcitonin receptor (Calc. R), and beta3-integrin in CQ-treated cultures.

To understand how CQ inhibits OC genesis, we first tested whether M-CSF signaling was altered by CQ treatment, because M-CSF and its receptor, c-Fms, drive early lineage development from BMMs and are essential for osteoclastic survival [23]. BMMs from WT and Tg2576 mice were serum-starved for o/n, and then incubated with M-CSF with or without CQ (5 microM) for various time. As shown in Fig 6A and 6B, M-CSF-induced phosphorylation of extracellular signal–regulated kinase (Erk1/2) and Akt/PKB (protein kinase B), but no difference was detected in BMMs treated with or without CQ, suggesting little to no effect on M-CSF signaling by CQ treatment.

We next examined RANKL signaling in WT- and Tg2576-BMMs in the presence or absence of CQ (5 microM), as RANKL and its receptor, RANK, are essential for the development of BMMs to mature OCs [3, 4], and RANKL activation of NF-κB, Erk1/2, and Akt is required for efficient osteoclastogenesis and OC activation [24, 25]. RANKL-driven phosphorylation of Erk1/2 appeared to be unchanged in both WT and Tg2576 BMMs treated with or without CQ (Fig 5C). However, RANKL-induced Akt phosphorylation in Tg2576-BMMs, but not WT-BMMs, was impaired by CQ treatment (Fig 6C and 6D). These results suggest that CQ had little to no effect on M-CSF-, integrin-, or RANKL-induced early signaling events (e.g., pErk1/2) in both WT- and Tg2576-BMMs. However, CQ selectively reduced RANKL-driven Akt phosphorylation in Tg2576-BMMs.

We further examined the late RANKL signaling events in WT- and Tg2576-BMMs in response to CQ treatment. WT- and Tg2576-BMMs were incubated with M-CSF and RANKL in the presence or absence of CQ (5 microM) for various days. NFATc1 induction/activation by RANKL was specifically examined, as it is an essential late signaling event for OC maturation and activation [25,26]. Note that the immunoreactive NFATc1 was induced in both WT- and Tg2576-BMMs after exposure to RANKL and M-CSF (Fig 6E and 6F), which migrated more rapidly probably caused by RANKL/M-CSF-induced activation of a calcium/calcineurin and dephosphorylation of NFATc (an indicator of NFATc1 activation)[25, 26]. Interestingly, the NFATc1 induction/activation was largely reduced in both WT- and Tg2576- cultures treated with CQ (Fig 6E and 6F). A more dramatically CQ inhibition was detected in Tg2576-BMMs, compared with that in WT-BMM cultures (Fig 6E and 6F). RT-PCR analysis also showed reduced expression of genes associated with OC maturation, including MMP9, Calcitonin Receptor (Calc. R), and beta3-integrin, in CQ-treated cultures (Fig 6G). In aggregate, these results suggest that CQ’s inhibition of OC maturation in Tg2576-BMMs might be due to its selective inhibition of RANKL-driven Akt phosphorylation and NFATc1 activation.

### CQ-decrease of intracellular iron levels and increase of ferriportin (FPN) in BMMs with more potency in Tg2576-BMMs

**Fig 7:**
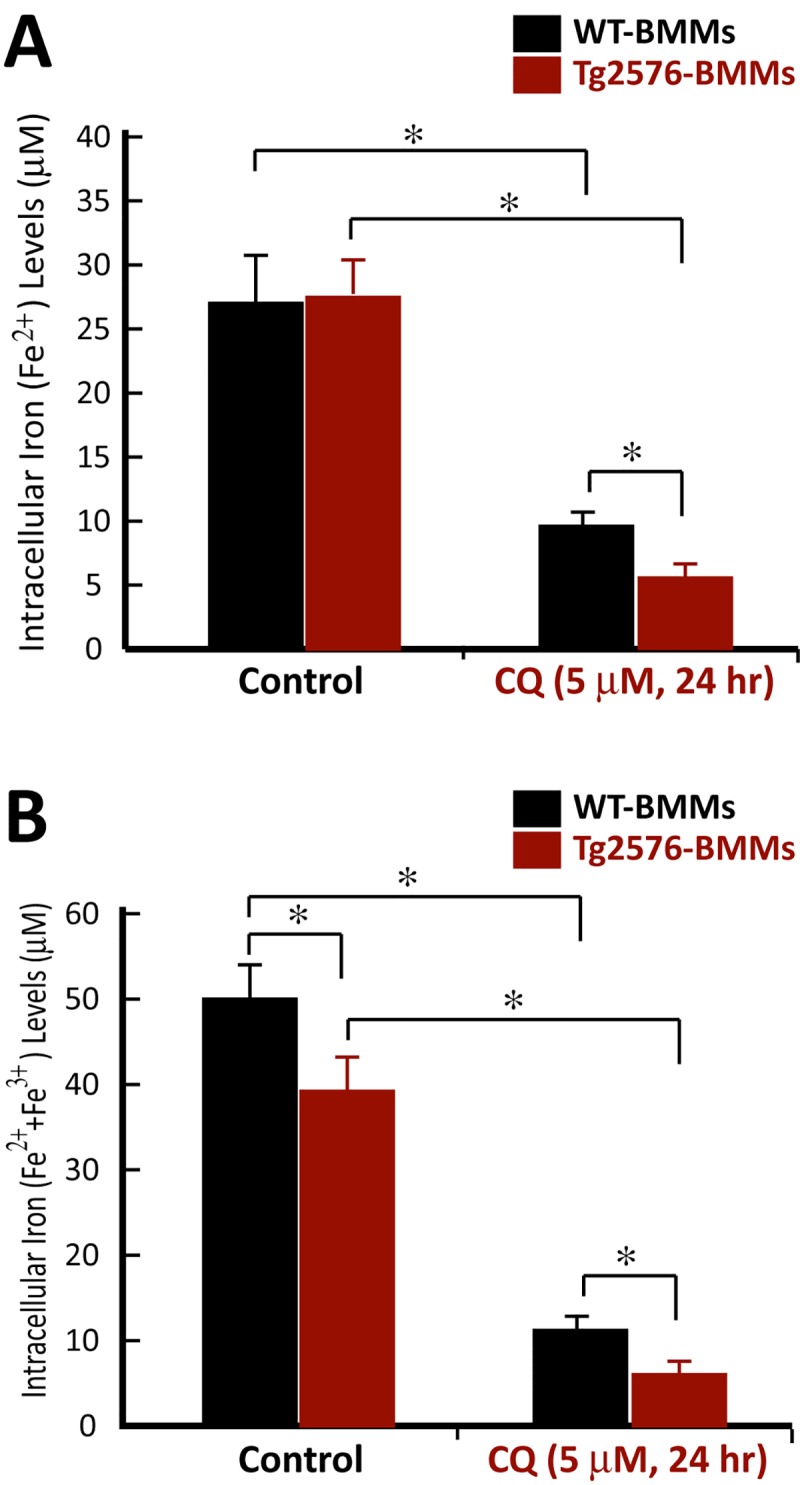
CQ reduction of intracellular iron levels in WT- and Tg2576-BMMs in vitro. BMMs from WT- and Tg2576 mice were incubated in the absence or presence of 5-microM CQ for 24 hours. Cells were harvested, and the intracellular total Fe2+ and Fe3+ concentrations were determined by Iron colorimetric assay (see Methods). The values of mean ± SD from 3 separate assays were shown. *, P < 0.05, significant difference.

The selective inhibitory effect on OC maturation in Tg2576-BMMs by both CQ and DFO lead to the speculation a differential iron chelation effect that may occur between Tg2576-BMMs and WT-BMMs. To test this speculation, we first compared CQ’s iron chelation effect between WT- and Tg2576-BMMs by measuring the intracellular iron (both Fe2+ and Fe3+) levels. The CQ treatment (5 microM, for 24 hrs) indeed reduced iron levels in both WT- and Tg2576-BMMs (Fig 7A and 7B). Interestingly, this effect was more dramatic in Tg2576-BMMs (Fig 7A and 7B), revealing a correlation between iron chelation and OC inhibition, and supporting the speculation.

**Fig 8:**
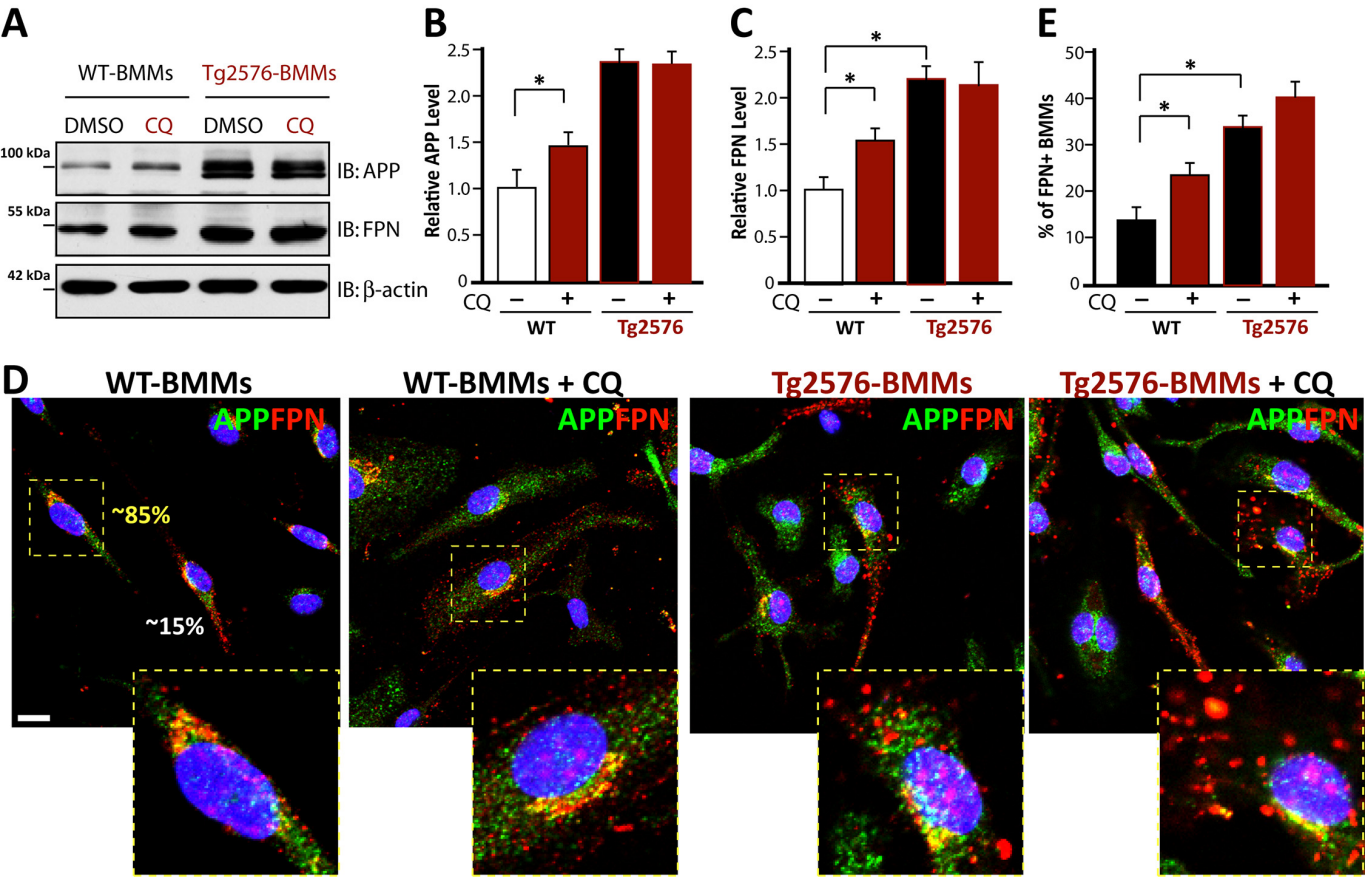
CQ regulation of APP and ferriportin (FPN) in BMMs. (A-C) Western blot analyses of WT- and Tg2576-BMMs treated with or without CQ (5 microM) using indicated antibodies. A, Representative blots. B, Quantification analysis of relative APP level (normalized by WT-BMMs, no CQ). C, Quantification analysis of relative FPN level (normalized by WT-BMMs, no CQ). (D-E) Immunostaining analysis of WT- and Tg2576-BMMs treated with or without CQ (5 μM) using indicated antibodies. D, Representative images. Inserts were amplified images. Scale bar, 10 mm. E, Quantification analysis. The % of BMMs with cell surface distributed FPN were presented. The values of mean ± SD from 3 separate assays were shown. *, P < 0.05, significant difference.

We then examined CQ’s effect on FPN in WT- and Tg2576-BMMs, because FPN is a key iron exporter that is highly expressed in macrophages [11], APP interacts with FPN, and APP is implicated in promoting FPN’s iron export activity [10]. Western blot analysis indicated that both FPN and APP proteins were increased in CQ-treated WT-BMMs (Fig 8A–8C). No obvious change in APP/APPswe and FPN levels was detected in Tg2576-BMMs treated with CQ. However, FPN was much higher in Tg2576-BMMs than that of WT-BMMs (Fig 8A–8C). These results suggest that both CQ treatment and expression of APPswe resulted in an increase of FPN level.

To further test this view, CQ treated or untreated WT-BMMs and Tg2576-BMMs were subjected to the co-immunostaining analysis of FPN and APP. As shown in Fig 8D, most WT-BMMs showed a partial co-localization of APP with FPN, which appeared to be distributed in the perinuclei region, but not in the cell surface (Fig 8D). Few WT-BMMs (~15%) showed high level of FPN in the cell surface (Fig 8E). Upon CQ treatment, the cell surface FPN was increased in both WT- and Tg2576-BMMs (Fig 8D and 8E). It is of interest to note that Tg2576-BMMs showed higher percentage of BMMs with cell surface-distributed FPN (Fig 8D and 8E). These results are in line with the Western blots, suggesting that by increasing cell surface FPN, CQ and APPswe may promote iron export activity and reduce intracellular iron levels.

## Discussion

**Fig 9:**
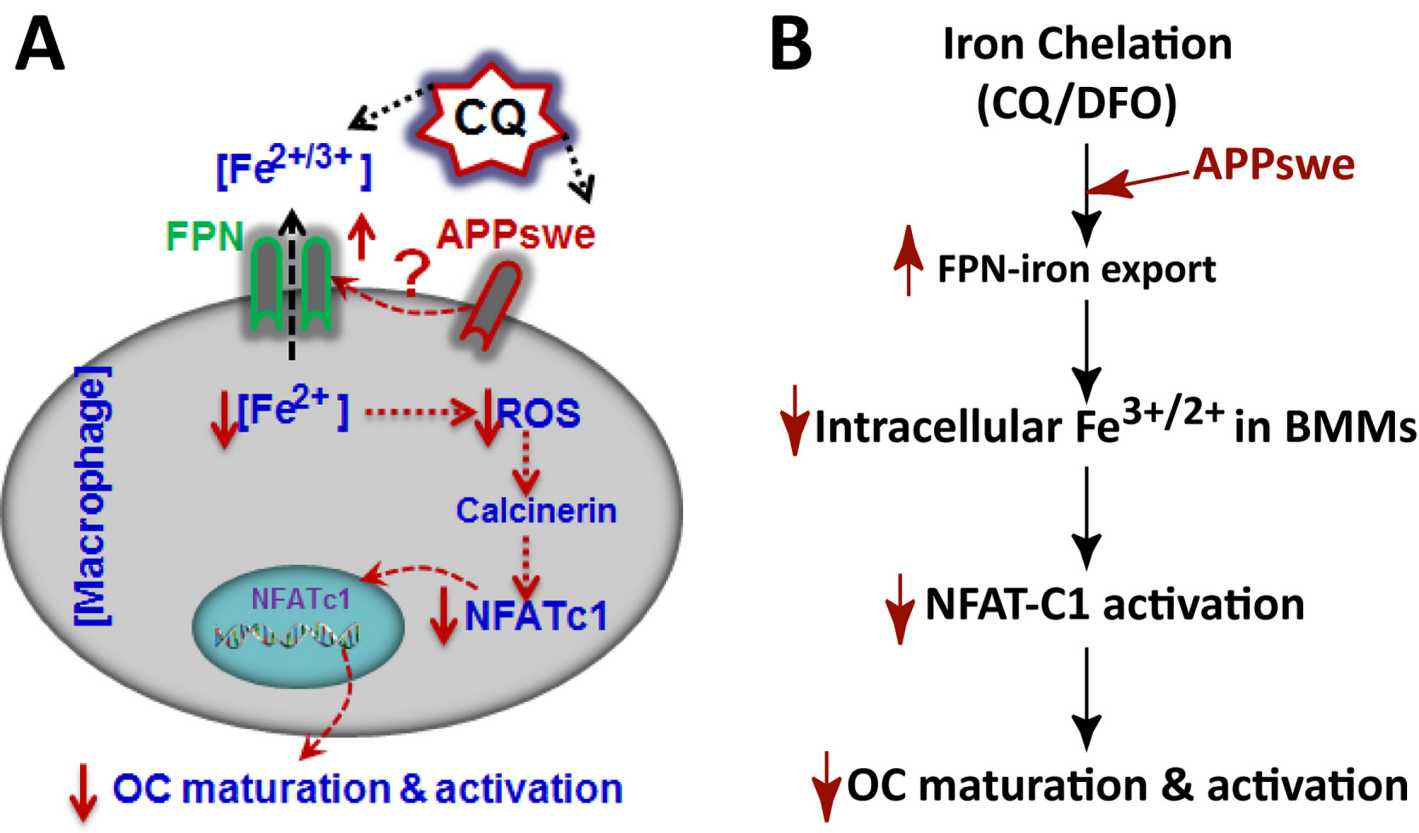
Illustration of a working model for inhibition of OC-genesis by iron chelation. Iron chelation by CQ or DFO inhibits OC maturation, which might be due to its role in increasing FPN-mediated iron exporting in BMMs, thus, reducing intracellular iron levels, impairing NFAT-C1 activation, and decreasing OC-maturation. This effect is enhanced by expression of APPswe in BMMs.

This study provides evidence that iron chelation in BMMs inhibits osteoclastogenesis and bone resorption. Based on our in vitro studies and in light of the literature reports, we proposed a working model depicted in Fig 9 to illustrate the possible underlying mechanism(s). In this model, CQ inhibits OC maturation and activation likely by increasing FPN iron exporter, thus reducing intracellular irons and iron involved calcinurin-NFATc1 activation. This work supports the view that intracellular iron in BMMs is critical to promote OC-genesis and activation, and suggests that CQ or other iron chelater(s) may be valuable therapeutic agent(s) in treating AD-associated osteoporotic deficit.

Elevation of intracellular iron levels in BMMs is believed to be critical for OC differentiation and activation [27, 28]. In vitro incubation of BMMs with iron donors enhances OC-differentiation [29]. In vitro treatment with hepcidin, a ligand of FPN that reduces FPN-mediated iron export, in BMMs or RAW264.7 macrophages, not only increases intracellular iron levels, but also facilitates OC differentiation [30]. Thus, it is conceivable that iron chelation would be a negative factor for OC-differentiation. Indeed, our studies suggest an OC-inhibition by iron chelators, CQ and DFO. It is noteworthy that CQ is often described as a zinc and copper (more than an iron) ion chelator. Thus, it is also possible that the chelation of zinc and copper ions, in addition to iron, contributes to CQ’s inhibitory effect on OC-genesis. It is also of interest to note that the CQ/DFO’ inhibitory effect on OC genesis was enhanced/sensitized by expression of APPswe in BMMs. In vitro treatment with low doses of both iron chelators, clinoquinol and DFO, could reduce OC differentiation in APPswe-expressing BMMs, but not WT-BMMs (Fig 5). In vivo treatment with CQ also selectively decreased TRAP+ OC cell numbers and OC-mediated bone resorption in mice with APPswe expression in OC-lineage cells (e.g., Tg2576), but not in WT-mice (Figs 1 and 2) or in mice with APPswe expression in OB-lineage cells (TgAPPswe-Ocn)(data not shown). Whereas these results reveal an increased potency of CQ or DFO in APPswe expressing BMMs, as compared with that of WT-BMMs, the exactly underlying mechanisms require further investigation.

How does iron chelation in BMMs suppress OC differentiation? This is likely due to iron chelation-induced reduction of free radicals or ROS (radical oxidant species) in OC-lineage cells. The intracellular Fe2+/3+ is a critical source for cellular ROS generation, and the ROS is found to be critical for OC differentiation and activation [28]. In viewing the literature, it is of interest to found that Fe2+/ROS is an activator of calcineurin-NFATc1 pathway that is essential for OC differentiation [27–30]. In agreement with this view were our observations that CQ has little effect on early signaling events induced by M-CSF, RANKL, or integrin (Fig 6G), but markedly reduced NFATc1 activation and OC-maturation (Fig 6).

Our studies suggest that CQ may be effective in treating AD-associated osteoporotic deficit in AD animal models, raising a question of whether CQ could act as a therapeutic agent in treating AD-associated osteoporosis in patients. It is noteworthy that CQ was used as an antiseptic drug to treat intestinal diseases. But, it was withdrawn from the market in early 1970s, because the association of sub-acute myeloptic neuropathy (a syndrome that involves sensory and motor disturbances in the lower limbs and visual changes) with CQ treatment was detected. However, in light of the recent metal hypothesis of AD [7], many scientists re-consider the possible use of CQ or CQ derivatives. Whereas our studies presented here support the re-use of CQ, this issue requires further examination by additional animal models and clinical trials.
